# Letter to the editor of Heliyon re: Bioinformatics-based prediction and screening of immunogenic epitopes of *Toxoplasma gondii* rhoptry proteins 7, 21 and 22 as candidate vaccine target

**DOI:** 10.1016/j.heliyon.2024.e32221

**Published:** 2024-06-04

**Authors:** Fariha Ayub, Haroon Ahmed, Tehreem Sohail, Khurram Shahzad, Figen Celik, Xu Wang, Sami Simsek, Jianping Cao

**Affiliations:** Department of Biosciences, COMSATS University Islamabad (CUI), Park Road, Chakh Shahzad, Islamabad, Pakistan; Department of Biosciences, COMSATS University Islamabad (CUI), Park Road, Chakh Shahzad, Islamabad, Pakistan; Department of Biosciences, COMSATS University Islamabad (CUI), Park Road, Chakh Shahzad, Islamabad, Pakistan; Department of Biosciences, COMSATS University Islamabad (CUI), Park Road, Chakh Shahzad, Islamabad, Pakistan; Department of Parasitology, Faculty of Veterinary Medicine, Firat University, Elazig, Turkey; National Institute of Parasitic Diseases, Chinese Center for Disease Control and Prevention (Chinese Center for Tropical Diseases Research), Key Laboratory of Parasite and Vector Biology, National Health Commission of the People's Republic of China, WHO Collaborating Center for Tropical Diseases, Shanghai, China; Department of Parasitology, Faculty of Veterinary Medicine, Firat University, Elazig, Turkey; National Institute of Parasitic Diseases, Chinese Center for Disease Control and Prevention (Chinese Center for Tropical Diseases Research), Key Laboratory of Parasite and Vector Biology, National Health Commission of the People's Republic of China, WHO Collaborating Center for Tropical Diseases, Shanghai, China; The School of Global Health, Chinese Center for Tropical Diseases Research, Shanghai Jiao Tong University School of Medicine, Shanghai, China

Dear Editor,

We really appreciate the painstaking appraisal of our manuscript by the worthy reviewer (s). Toxoplasmosis is a zoonotic disease that is caused by *Toxoplasma gondii* and most common parasitic infection among human and animals [1]. *T. gondii* is an obligate intracellular coccidian parasite and is responsible of causing infections in many homeothermic species and forms the cyst in tissues [2]. It has been estimated that in human *T. gondii* responsible of causing infection in more than one third of the population [1]. However, on worldwide basis the frequency of occurrence of *T. gondii* antibodies among cattle reaches up to the 92 %, depending upon the investigated region [3]. The data on occurrence of this protozoan or its DNA in tissues of bovine have been published under natural and experimental conditions [[3], [4], [5], [6], [7]]. In past published literature is avaliable on toxoplasmosis outbreaks caused due to the consumption of meat from infected cattle [8]. In endemic regions, the importance of this food in lifecycle stages of human toxoplasmosis can not be ignored [9]. Therefore due to the severity of this diseases among immunocompromised individuals and pregnent womens, transmission dynamics of the protozoans diseases via consumption of infected meat to be of great importance for public health [2,6]. Therefore, *T. gondii* infection in cattle may not cause clinical signs but it is known to cause abortion among these animals [2,10]. On world wide basis, toxoplasmosis is the 4th most common foodborne infection [11] and the contaminated meat of infected animals is a potenial source of infection in humans, previous reports on *T. gondii* infection among cattle are therefore mainly focused on interest related to public health [6]. A comprehrensive collaborative study was conducted in United Kingdom, Romania, Italy and Netherlands (n = 100/country) to investigate the live parasites and their DNA inthe samples from diaphragms (200 g). Finally, *Toxoplasma gondii* DNA was reported in tissues of 13 out of 401 cattle [7]. Therefore, it would not be correct to say that toxoplasmosis is not important in cattle.

GenBank stands as an extensive public repository housing nucleotide sequences alongside pertinent bibliographic and biological annotations, administered by the National Center for Biotechnology Information (NCBI), a division under the National Library of Medicine (NLM) [12]. It serves as a pivotal resource for numerous scientific endeavors, offering access to genetic sequence data across a spectrum of applications, encompassing genome analysis, evolutionary research, gene expression analysis, functional annotation, and ongoing updates. Specifically, in terms of *T.* *gondii* research, GenBank has been used for protein sequence retrievals as indicated by many research articles [[13], [14], [15], [16]]. Therefore, it is not appropriate to consider NCBI database less significant.

We appreciate about the comment regarding the use of ElliPro tool as it can also be used for linear B cell epitope prediction and we also appreciate the recommended tools such as SVMTriP, ABCpred, BepiPred 2.0, LBtope, etc. which will definitely be considered in future studies. Nonetheless, ElliPro tool is equally useable for continuous and discountinuous B cell epitope predictions as argued by Soria-Guerra et al. [17] in Table 3 and by Ren et al. [18].

Regarding the use of the IEDB tool for immunogenicity prediction, we want to clarify that the inclusion of the IEDB immunogenicity tool does not compromise the validity or integrity of the remaining content in the article. As it has been stated by Soria-Guerra et al. [17] in section 4 titled “Bioinformatics tools for predicting potential B cell binding-epitopes” that “Continuous B cell epitope prediction is very similar to T cell epitope prediction, which has mainly been based upon the amino acid properties such as hydrophilicity, charge, exposed surface area and secondary structure”. However the predicted results by http://tools.iedb.org/immunogenicity/tools can be justified for designing of the vaccination process. In our opinion, use of IEDB tool does not compromise our findings. However, it is agreed that use of suggested tool should be preferred in upcoming studies.

During our research, we conducted a comprehensive examination of the allergenicity and antigenicity properties of the proteins, as outlined in section 2.4 of the methodology. The results of this investigation are detailed in section 3.3. It is pertinent to address the issue of solubility. However, we observed some studies such as [[19], [20], [21]] in which solubility testing was not considered a mandatory requirement.

During the current study, our work deals with determination of immunogenic epitopes by using humoral associated epitopic regions instead of cell mediated immunity as reported in previous studies [[21], [22], [23], [24]]. We appreciate the comment regarding the cell mediated immunity which can be designed for the epitope prediction in the future studies.

## CRediT authorship contribution statement

**Fariha Ayub:** Writing – review & editing, Writing – original draft. **Haroon Ahmed:** Writing – review & editing, Writing – original draft. **Tehreem Sohail:** Writing – review & editing, Writing – original draft. **Khurram Shahzad:** Writing – original draft. **Figen Celik:** Writing – original draft. **Xu Wang:** Writing – review & editing. **Sami Simsek:** Writing – review & editing, Writing – original draft. **Jianping Cao:** Writing – review & editing.

## Declaration of competing interest

The authors declare that they have no known competing financial interests or personal relationships that could have appeared to influence the work reported in this paper.
